# Disentangling diagnostic object properties for human scene categorization

**DOI:** 10.1038/s41598-023-32385-y

**Published:** 2023-04-11

**Authors:** Sandro L. Wiesmann, Melissa L.-H. Võ

**Affiliations:** Department of Psychology, Johann Wolfgang Goethe-Universität, Theodor-W.-Adorno-Platz 6, 60323 Frankfurt Am Main, Germany

**Keywords:** Human behaviour, Object vision

## Abstract

It usually only takes a single glance to categorize our environment into different scene categories (e.g. a kitchen or a highway). Object information has been suggested to play a crucial role in this process, and some proposals even claim that the recognition of a single object can be sufficient to categorize the scene around it. Here, we tested this claim in four behavioural experiments by having participants categorize real-world scene photographs that were reduced to a single, cut-out object. We show that single objects can indeed be sufficient for correct scene categorization and that scene category information can be extracted within 50 ms of object presentation. Furthermore, we identified object frequency and specificity for the target scene category as the most important object properties for human scene categorization. Interestingly, despite the statistical definition of specificity and frequency, human ratings of these properties were better predictors of scene categorization behaviour than more objective statistics derived from databases of labelled real-world images. Taken together, our findings support a central role of object information during human scene categorization, showing that single objects can be indicative of a scene category if they are assumed to frequently and exclusively occur in a certain environment.

## Introduction

Humans can quickly and effortlessly make sense of and categorize visual scenery^1,2^. Within a glimpse, we can assess whether a scene is indoor or outdoor (superordinate-level categorization) and, more specifically, a forest, bedroom, or shop (basic-level categorization)^3,4^. This information can be highly relevant for subsequent interactions with the scene (e.g. action planning, object search or navigation^5^). Despite the ease of human scene categorization and its relevance in our everyday life, the exact scene features that inform this task are not yet fully understood. In the past decades, most theories of human scene categorization have either taken an object-centred or scene-centred approach^6^, the former explaining scene categorization through the recognition of single objects that are assumed to be diagnostic of the scene category^7,8^ and the latter focussing on global scene properties such as spatial layout information that are independent of object identities^9,10^. Note, however, that these different approaches must be understood as *focussing* on either type of information. Scene perception is usually conceptualized as comprising both scene- and object-level processing, and several dual-pathway approaches exist^11–13^.

While global scene properties are computationally useful^6,9,10,14,15^ and explain neural responses in scene-selective brain regions^16,17^, their sufficiency for human scene categorization is to question. First, most computational models based on global scene properties only consider outdoor scenes with varying spatial layout^18^. Indoor scenes, on the other hand, usually have a similar spatial layout (limited openness, expansion, and depth) and mostly vary in their category-defining objects (e.g. a desk vs. a dinner table). Performance of most computational classifiers based on global scene properties therefore drops significantly on typical indoor scenes^18^. Second, stimuli reduced to global scene properties have been shown to be unrecognizable by human observers in a free-response task^19^. Moreover, these texturized stimuli yield categorization performance far below unmanipulated images, and better performance is usually correlated with higher rates of object recognition in these textures^20^. Human-rated estimates of spatial layout are further poor predictors of scene categories^4^. It therefore seems unlikely that global scene properties *alone* are sufficient to explain the effortless scene categorization ability of humans.

Object information, on the other hand, has traditionally been suggested as a plausible candidate in the search for scene features that drive human scene categorization^7,8^. Intuitively, we would assume that we recognize a kitchen as such because we see a fridge and a stove in it. After all, we are surrounded by and interact with objects ceaselessly every day, and we even define certain scene categories using single key objects they contain (e.g. bedrooms, forests). Importantly, we can use these key objects to make accurate assumptions about a scene’s category. When helping a friend move houses, we can guess that the “living room” box goes into the room with the couch and not the one with the stove. These associations of which objects belong together and form a scene seem to be deeply ingrained and are formed early on^21,22^.

Different lines of evidence support the notion that object information is central during scene categorization. Behaviourally, we are worse at categorizing scenes when objects are removed from them^23^ or semantically inconsistent objects are presented^11,12,24,25^. This is underlined by neural findings showing that scene-selective brain areas are also activated by single objects^26^, and their activity is modulated by object properties^27^. Computational scene classifier have been shown to benefit from the inclusion of object detectors, which lead to significant improvements in classification performance especially for indoor scenes^28,29^.

Assuming that object information plays an important role in human scene categorization opens up three questions that we sought to answer with the series of experiments presented in this article. First, we assessed whether human observers are indeed able to categorize scene images reduced to single objects only. Several classic theories of human scene categorization propose that this is possible: Friedman^8^ suggests that certain objects “are diagnostic with respect to instantiating or verifying a frame of reference” and “should be sufficient, but not necessary, for frame instantiation” (p. 325). Similarly, Biederman^7^ argues that “[o]ne route to a schema is through an initial identification of one (or more) of the more discriminable objects in a scene” (p. 239).

Second, we used extensive human ratings to identify the exact object properties that make an object diagnostic for human scene categorization. Several qualities of objects have been suggested to be relevant for categorization: Friedman^8^ argued that “obligatory” objects are diagnostic which she defined based on a high occurrence probability in a scene and strong thematic relation to a scene. This notion is closely related to other definitions of diagnostic objects based on occurrence frequency^30^ and the observation that “highly contextual” objects or “signature objects” drive activation in brain areas related to scene perception^23,31^. We tested this claim by explicitly assessing object frequency for each scene category and using a stimulus set with a certain variance of typicality across objects. On the other hand, Biederman^7^ only considered “more discriminable” objects as diagnostic and, importantly, argued that “processing of the first object(s) would be independent of the processing of other objects” (p. 239). We tested this by using both very well visible, large objects in a scene but also smaller, less salient objects in the periphery. Furthermore, Biederman proposed that “once an object is identified in a scene, we may quickly know the kind of company it keeps” (p. 239). This is closely related to the notion of anchor objects^32,33^. Anchor objects (e.g. a stove) are defined as large, usually stationary objects conveying spatial predictions about smaller, so-called local objects typically found in their proximity (e.g. a pan on top of the stove). We assessed whether anchor objects are also diagnostic by explicitly collecting ratings of anchorness in Experiment 1 and disentangling potentially diagnostic features of anchor objects (predictiveness of other objects, size, movability) in Experiment 2. Alternatively, large and stationary objects may also be diagnostic because they are space-defining and are crucial for maintaining mental representations of scenes^34,35^. To further rule out the possibility that single objects are simply diagnostic because they allow for an estimation of global scene information (e.g. openness, expansion, depth)^9,10^, we specifically assessed whether our single objects conveyed implications of scene size and spatial layout. Lastly, a higher-level approach to human scene categorization suggests that scenes are not categorized using visual features such as objects per se, but rather by the actions that can be performed in a scene (i.e. the scene’s functions)^36,37^. We therefore included measures assessing how often an object is used within a scene and whether it gives rise to action possibilities (e.g. a stove gives rise to the action *cooking*).

Third, after identifying object specificity and frequency as the core object properties driving human scene categorization based on single objects, we further compared the utility of human ratings and database-derived measures of object properties in explaining human scene categorization performance. The conclusions from this comparison may not only guide authors in the design of future studies, they also help us better understand the cognitive mechanisms involved in human scene categorization.

Taken together, our findings corroborate object-centred theories of human scene categorization, showing that human observers can indeed perform meaningful scene categorizations based on single objects only. This ability is best explained by participants’ ratings of assumed object frequency and specificity for the target scene category and not as much by objective, database-generated measures. We discuss implications of these findings for scene-centred and object-centred theories of human scene categorization, for proposals of the relevant properties that make an object diagnostic and for future studies seeking to assess object diagnosticity in a scene categorization task.

## Experiment 1

In Experiment 1, we assessed whether humans were able to correctly categorize scenes reduced to single objects as proposed by object-centred theories of human scene categorization^7,8^. We cut-out objects from real-world scene photographs, pasted them on a uniform grey background and let participants categorize them in a 16-AFC task. After the experiment, we collected ratings regarding the objects’ diagnosticity and anchorness to assess whether these object properties predicted scene categorization accuracy. Diagnosticity refers to an objects’ ability to predict the scene category of the context it usually occurs in and goes back to the proposal of Friedman^8^ and the first route suggested by Biederman^7,30^. On the other hand, anchorness refers to the ability of certain objects (so-called anchor objects)^32,33^ to predict the occurrence and position of other, related objects (so-called local objects). For example, a sink is considered an anchor object because it usually predicts the occurrence and position of soap (on the sink, next to the tap), towel (lateral to the sink on the wall) and mirror (above the sink on the wall). Anchor objects have been shown to be crucial for object search in both 2D^32^ and 3D^38^. Here we tested whether they are also related to our ability to categorize real-world scenes.

### Method

#### Participants

Seventy-one volunteers participated in Experiment 1 for course credit. A simulation-based power analysis run on pilot data by five participants using the *mixedpower* package for R^39^ showed that a sample size of 30 was sufficient to reach power > 0.80 in the models testing the effects of diagnosticity and anchorness on basic-level categorization accuracy. However, since the experiment was carried out as part of a psychology undergraduate class in which students were required to participate in a certain number of studies for course credit, we collected more data. We excluded data of one participant due to technical problems during data collection and another six participants who yielded categorization performance more than two standard deviations below the group average, leaving data of 64 participants for analysis in Experiment 1 (51 female, 12 male, 1 not specified; 18–35 years old, *M* = 21.6 years). All volunteers had normal or corrected-to-normal vision, were unfamiliar with the hypotheses of the study and the stimulus material, and gave informed written consent before participating in the study. The experimental protocols of all experiments presented in this article were approved by the Goethe University’s Human Research Ethics Committee (ethics approval ID# 2014-106R1) and followed the Goethe University’s guidelines for experimental studies with human participants.

#### Stimuli

**Figure 1:**
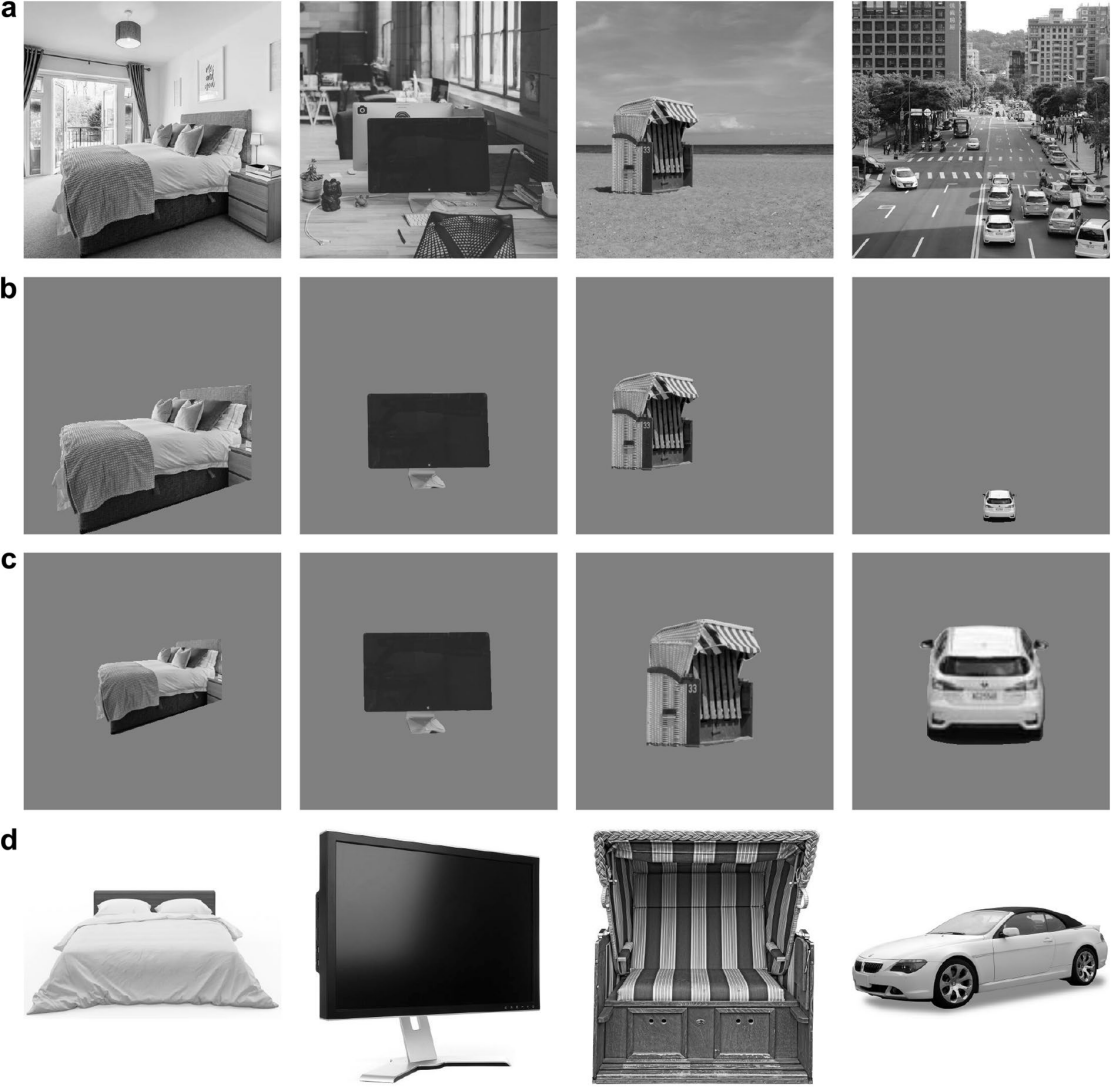
Examples of stimuli used. (**a**) Unprocessed base stimuli (not used in the experiments). (**b**) Stimuli reduced to a single object cut out from the original scenes as used in Experiments 1 and 2. (**c**) Stimuli reduced to a single resized and centred object cut out from the original scenes as used in Experiment 3. (**d**) Stimuli showing a prototypical image of the object as used in Experiment 4. All images from pixabay.com except for bed image in (**d**) by alexandercho, Freepik.com.

We used 240 black and white photographs of indoor and outdoor scenes (see Fig. 1a) comprising 16 different basic-level categories (indoor: bathroom, bedroom, kitchen, living room, classroom, office, restaurant, shop; outdoor: beach, desert, forest, mountain, city, highway, playground, train station; 15 stimuli per basic-level category). All stimuli were converted to grey scale, resized to 512 × 512 pixels, and matched in typicality based on human ratings^20^. For each scene, we hand-labelled several well visible objects using the *LabelMe* online tool^40^. We then replaced the scene around each annotated object with a uniform grey background and had three independent observers name the presented objects, rate how typical and diagnostic they were for the respective scene category, and identify whether they were anchor objects. For our final stimulus set, we selected objects that were easily recognized, rated as typical exemplars and yet showed a certain variance across diagnosticity and anchorness ratings that was comparable across scene categories. The resulting stimulus set consisted of 240 unique scene images reduced to a single object on a grey background. In Experiments 1 and 2, the object was always reproduced in the same size and position as in the original scene (see Fig. 1b).

#### Apparatus

Experiment 1 was conducted online using the *jsPsych* library^41^. Participants were required to use a computer or laptop with a screen size of at least 14” and a physical keyboard. Stimuli were resized on screen using a built-in calibration feature of *jsPsych*, thus the absolute size of the displayed stimuli was the same for all participants (ca. 14 × 14 cm), irrespective of physical screen size. Note, however, that we were not able to control for viewing distance.

#### Procedure

All instructions were presented on screen in written form. Participants performed speeded categorizations of scenes from 16 different categories that were reduced to single objects. Each trial followed the same structure: (1) presentation of a fixation cross (1000–1600 ms), (2) presentation of a randomly selected stimulus (1000 ms), (3) superordinate-level categorization (indoor vs. outdoor, until response), (4) basic-level categorization (one out of eight indoor or outdoor categories, depending on the participant’s superordinate-level categorization, until response), and (5) a confidence rating (six-level Likert scale from 1—*not confident at all* to 6—*very confident*). Participants performed the categorization tasks and the confidence rating using the number keys 1–8 on their keyboard. We instructed them to respond as quickly as possible but to prioritize accuracy over speed. When participants were not able to identify the scene category of the stimulus, they were encouraged to select the category they thought was most likely correct. Experiment 1 comprised 240 trials (plus 16 practice trials) with pauses after each block of 40 trials during which participants received visual feedback on the mean accuracy of their categorizations. After the main experiment, participants saw the same stimuli again and rated their diagnosticity and anchorness on scales from 1 (*not diagnostic at all* or *not an anchor*) to 6 (*very diagnostic* or *strong anchor*). Definitions of diagnosticity and anchorness (see Online Supplement) were presented along with examples at the beginning of each rating block.

#### Analysis

All analyses were carried out in *RStudio*^42^ using the *tidyverse* package^43^. Data from practice trials were discarded, leaving a total of 15,360 observations for analysis. We analysed superordinate- and basic-level categorization accuracy data with generalized linear mixed effect models (GLMMs) using the *lme4* package for R^44^. The random effects structure of all models was set maximal^45^, including a random intercept for participants (64), basic-level scene categories (16), and items (the 240 individual base stimuli). Random slopes for the respective predictors specified in each model were added within participants and basic-level scene categories (see Online Supplement for model specifications).

## Results and discussion

**Figure 2:**
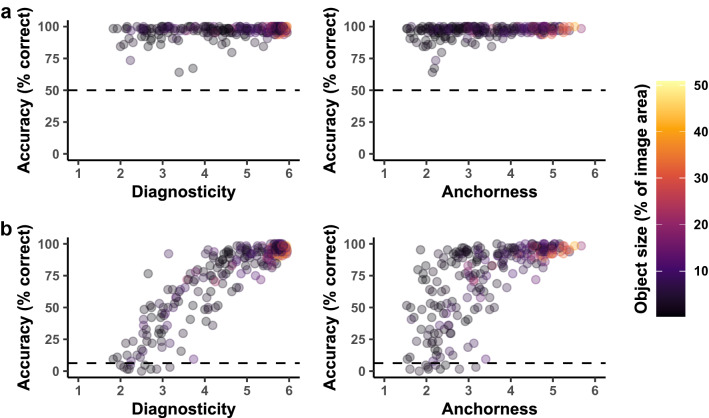
Observed (**a**) superordinate-level and (**b**) basic-level scene categorization accuracy in Experiment 1 as a function of diagnosticity, anchorness and object size on the screen. Points represent individual images averaged across participants. The dashed lines indicate the expected chance level in the superordinate-level (50%) and basic-level scene categorization task (6.25%).

Observed scene-categorization accuracies are summarized in Fig. 2. Performance in the superordinate-level categorization task (Fig. 2a) was at ceiling with a mean categorization accuracy of 96.6% (indoor scenes: 96.2%, outdoor scenes: 97.0%) and most stimuli yielding accuracies above 90%. At the basic level (Fig. 2b), there was still a high proportion of stimuli with near-perfect categorization accuracies, but the variance was overall higher with a mean categorization accuracy of 73.3% (indoor scenes: 71.1%, outdoor scenes: 75.6%). Participant-rated diagnosticity and anchorness were positively correlated (*r* = 0.55). Higher ratings of diagnosticity were related to better average task performance, showing that participants were able to judge whether objects are indicative of a scene’s category. Anchorness also showed a positive, albeit weaker, relation with accuracy. Objects rated with an average anchorness of 5 or more did not yield basic-level categorization accuracies below 90%. However, we also observed high accuracies for objects with low anchorness ratings, implying that anchorness is sufficient but not necessary for correct categorization.To test whether participant-rated diagnosticity and anchorness could predict categorization performance, we compared GLMMs containing different subsets of predictors with Likelihood-Ratio-Tests (see Supplementary Table S1). We first set up models only containing the covariates object size (percentage of image covered) and eccentricity (measured as the Euclidian distance between the object’s centroid and the centre point of the image; see Model M1 in Supplementary Table S1). Note that we did not include an interaction of object size and eccentricity as it did not improve model fit significantly. We then added either participant-rated diagnosticity (Model M2) or anchorness (Model M3) to the models containing object size and eccentricity to assess their relevance individually. In a last step, we entered both diagnosticity and anchorness into one model (Model M4). Note that, at the superordinate level, we restricted correlations of random slopes within scene categories to zero to ensure that models converged on meaningful results.

As expected, diagnosticity (M2 vs. M1) significantly improved model fits both at the superordinate, χ^2^(6) = 30.44, *p* < 0.001, and basic categorization level, χ^2^(9) = 654.00, *p* < 0.001. On the other hand, anchorness (M3 vs. M1) only improved model likelihood at the basic, χ^2^(9) = 114.80, *p* < 0.001, but not at the superordinate categorization level, χ^2^(6) = 7.55, *p* = 0.273, and the effect of anchorness on categorization accuracy was descriptively smaller compared to that of diagnosticity. Taken together, the results of these models imply that both participant-rated diagnosticity and anchorness are, individually, relevant predictors of human scene categorization, at least at the basic categorization level.

To assess whether diagnosticity and anchorness explained accuracy independently, we entered both predictors into a single model (M4) and compared it to a model only containing diagnosticity (M2). Adding anchorness did not significantly improve model likelihood, neither at the superordinate, χ^2^(7) = 8.78, *p* = 0.269, nor at the basic level, χ^2^(11) = 17.46, *p* = 0.095. And while the difference in model fits was close to significance at the basic level, the estimate for anchorness was not significantly different from zero in the models that contained it (see Supplementary Table S1, Model M4). We assume that this is primarily because most anchor objects are diagnostic, yet there are also diagnostic non-anchors, thus making diagnosticity the better predictor of categorization accuracy when both predictors are entered into the same model (see also Fig. 2). The fact that anchorness does not improve model fit or yield estimates different from zero further implies that anchorness does not contribute relevant information beyond that already contained in diagnosticity. It is therefore likely that not anchorness itself, the quality to predict the presence and location of other objects, but the co-occurrence of diagnosticity and anchorness in anchor objects drives the isolated effect of anchorness we observed in Model M3.

In summary, we show that participants can correctly categorize scenes reduced to single objects, and they can also assess whether objects are predictive of a scene’s category. Our results further imply that anchor objects are useful for human scene categorization, however not necessarily because anchorness itself is a useful object property for the categorization process, but because anchor objects happen to also be diagnostic. Diagnosticity and anchorness seem to co-occur in anchor objects, but there are also diagnostic objects that are not anchors (see Fig. 2). Participant-rated diagnosticity therefore turns out to be the best predictor of scene categorization. The question we move to next is which object properties constitute diagnosticity.

## Experiment 2

In Experiment 1, we showed that most of our scenes could still be categorized correctly when reduced to just a single object. Participant-rated measures of diagnosticity and anchorness were predictive of categorization accuracy, but anchorness did not explain any variance beyond that explained by diagnosticity. In Experiment 2, we further disentangled the concept of diagnosticity and identified object properties that are indicative of a scene’s category using extensive ratings of our stimuli. We further replicated our findings using reduced presentation times to avoid ceiling effects in our data and probe the utility of single objects under more controlled viewing conditions. Lastly, we compared the utility of participant-rated and more objective, database-derived measures of different object properties for predicting human scene categorization based on single objects.

### Method

The method of Experiment 2 was identical to Experiment 1, except for the deviations noted below.

#### Participants

Seventy-one undergraduate students volunteered in Experiment 2. Separate power analyses for the ten object properties we were interested in assessing showed that a sample size of 62 was sufficient to reach power > 0.80 for most variables. We excluded data of six participants who did not complete both parts of the experiment, one participant who did not fulfil participation requirements, and two participants who yielded categorization performance more than two standard deviations below the group average, leaving data of 62 participants for analysis (50 female, 12 male; 18–30 years old, *M* = 21.3 years). None of the participants of Experiment 1 participated in Experiment 2.

#### Procedure

The procedure of Experiment 2 was identical to Experiment 1, except we presented stimuli for only 50 ms. For five participants, we additionally assessed whether the single objects were still recognizable with the 50 ms presentation time by adding an answer prompt after each trial asking the participants to name the object in a free-response format. After the main experiment, participants completed a second part in which they saw each image again and rated it on ten different rating items (see Table 1) using a scale from 1 (*do not agree at all*) to 6 (*fully agree*). The ratings were presented in blocks. For each rating item, the order of the presented stimuli within the rating block was randomized. No instructions or examples regarding the rating items were given in Experiment 2.

**Table 1:** Translations of the rating items used in Experiments 2, 3 and 4.

Abbreviation	Item
Diagnosticity	One can unambiguously infer the scene category X from the presented object.
Specificity	The presented object only occurs in scene category X.
Frequency	In scenes of category X, one can usually find the presented object.
Predictiveness	Using the presented object, one can infer which other objects are around it.
Usage frequency	The presented object is often used in scenes of the category X.
Object size	The presented object is big.
Scene layout	One can infer the spatial layout of the scene based on the presented object.
Scene size	One can infer the size of the scene based on the presented object.
Affordance	One can infer which actions can be carried out in the scene based on the presented object.
Movability	The presented object can be moved easily.

The “X” was replaced with the category label of the scene presented on the given trial.

Ratings of the diagnosticity item were only collected in Experiment 2.

#### Analysis

Data from practice trials were discarded, leaving a total of 14,880 observations for analysis. In all further analyses, we inverted ratings of movability as we assumed that stationary objects are more diagnostic than movable objects^32^. To identify object properties among the ratings we collected that are relevant for human scene categorization, we used a *Lasso* model selection procedure^46^ implemented in the *glmmLasso* package for R^47^. This procedure determines the relevant predictors in a model while balancing model likelihood and model sparsity. We first entered all rating items (except for diagnosticity to avoid predictors conveying redundant information) and the scaled covariates object size and eccentricity. The Lasso procedure then applies a “weight” to all regression coefficients, thus starting from a completely sparse model in which all predictors are constrained to zero. The parameter constraints are then slowly relaxed, thus making relevant predictors emerge until an optimal model is found for which the Bayesian Information Criterion (BIC)^48^ is minimal. Since the Lasso procedure is computationally demanding, we only ran models with random intercepts for participants (62), basic-level scene categories (16), and items (240). After identifying the relevant predictors for each model, we confirmed the fit of these models by including random slopes for the respective predictors within participants and basic-level scene categories (see Online Supplement for final model specifications).

In a second step, we compared the rating-based model identified by the Lasso procedure with models including more objective, database-generated measures of object properties. We used the statistics provided by the ADE20K database^49^ as it is one of the most thoroughly annotated image databases available and its categories largely overlap with the SUN database from which we obtained most of our base stimuli. In addition, we hand-labelled a total of 400 of our base stimuli (25 per basic-level category) using the *LabelMe* online tool^40^ to derive measures specifically for the subset of categories and stimuli we used in our experiments. In both databases, we calculated specificity of object X for scene category Y as the probability of the scene category Y given the presence of object X. In other words, we divided the number of occurrences of object X in scene category Y by the total number of occurrences of object X in the database. As specificity necessarily varies with the inclusion of additional distractor categories containing the target object, we calculated a second specificity measure for the ADE20K database using only a subset of the 16 scene categories we used in our experiment (and their synonyms for a total of 50 categories). We defined frequency of object X in scene category Y as the proportion of scenes in category Y containing object X. Note that, analogously to the rating item *Frequency* we used in the experiment (see Table 1), we did not consider the number of occurrences of object X within single scenes of category Y (e.g. the occurrence of multiple chairs within a restaurant scene), we merely counted how many scenes of category Y contain at least one object X.

## Results and discussion

**Figure 3:**
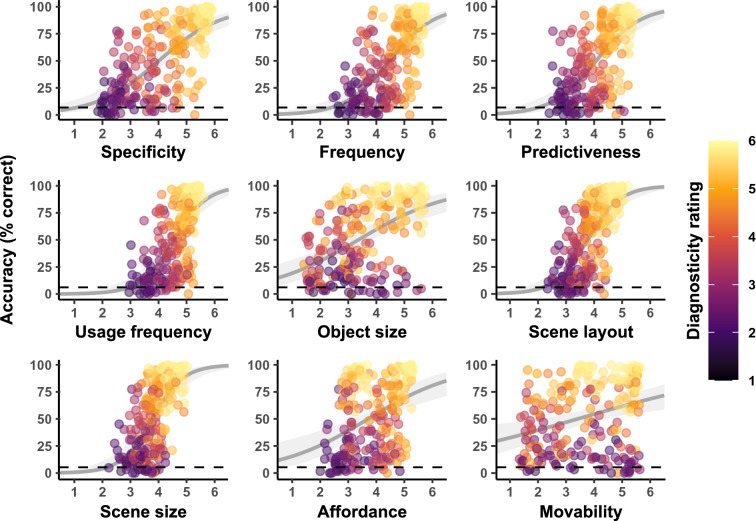
Observed basic-level scene categorization accuracy in Experiment 2 as a function of different ratings of object properties. Points represent the respective rating and accuracy averaged across participants for each individual image. Smoothed lines are fixed-effect binomial approximations for illustrative purposes only. The dashed lines indicate the expected chance level in the basic-level scene categorization task (6.25%).

Figure 3 summarizes the observed basic-level categorization accuracy per image as a function of the different object property ratings. Note that we focus our analysis on basic-level categorization accuracy as there was still a strong ceiling effect at the superordinate level (see Supplementary Fig. S1). Across images and participants, the mean superordinate-level and basic-level categorization accuracy was 86.9% (indoor: 84.4%, outdoor: 89.4%) and 52.6% (indoor: 45.0%, outdoor: 60.2%), respectively. Even with the presentation time of only 50 ms, most objects were still recognizable (see Supplementary Fig. S2) and yielded average scene categorization accuracy numerically above chance (more than 90% of objects at the superordinate and 85% at the basic level). This implies that single objects are still useful for scene categorization even under more constrained viewing conditions, and that object information can be extracted from stimuli quickly^50^. Upon inspection, almost all object property ratings showed a positive relationship with diagnosticity and categorization accuracy (see Fig. 3). This correlation was strongest for specificity, frequency and predictiveness, whereas participant-rated object size, action affordance, and object movability showed the weakest links.

To identify the exact object properties that were predictive of correct basic-level categorization, we used a penalized-regression variable-selection procedure (Lasso) for GLMMs^46^. This procedure identified a model containing the predictors specificity, frequency, usage frequency and predictiveness along with object size on screen and eccentricity as optimal (see Supplementary Table S2). However, a second, sparser model yielded an almost identical BIC (15,250.43 and 15,250.48, respectively). This model only included the predictors specificity, frequency, and object size on screen. Since we ran the Lasso procedure with random intercept-only models due to the high computational demands of the procedure, we decided to run both models again with the respective maximal random-effects structure^45^ and with both covariates for object size and eccentricity. A comparison of these models using a Likelihood-Ratio Test showed that the data did not fit the simpler model significantly worse than the optimal model identified by the Lasso procedure, χ^2^(28) = 32.54, *p* = 0.249. We therefore concluded that specificity and frequency of an object are the most relevant predictors of human scene categorization based on single objects.

To better understand which object properties drive human scene categorization for different scene categories, we repeated the Lasso procedure using subsets of indoor and outdoor scenes only (see Supplementary Table S3). Interestingly, for indoor stimuli, the Lasso procedure identified a model that, once again, contained significant predictors for object size, eccentricity, specificity and frequency, but that also contained (non-significant) predictors for the ratings of predictiveness, estimated object size and movability (inverted). These additional predictors are noteworthy as they measure object properties that are part of the definition of anchor objects: Anchor objects are assumed to be large, usually stationary objects that are predictive of other, smaller objects^32^. Note, however, that none of the three additional predictors was significant in this model and they all explained very little variance, similar to the single-item measurement of anchorness in Experiment 1. The emergence of these predictors for indoor stimuli is nonetheless of interest because the Lasso procedure we ran on the subset of outdoor stimuli only identified specificity and frequency as relevant predictors. Taken together, these results, in conjunction with the two similar models we identified using the Lasso procedure on the complete dataset, imply that specificity and frequency are the core object properties enabling human scene categorization. Depending on the scene category (indoor vs. outdoor stimuli), other, more qualitative object properties might contribute as well, but their influence is comparably small.

After identifying object frequency and specificity as the most relevant object properties for human scene categorization, we decided to estimate these predictors using more objective, database-generated measures. Both specificity and frequency are by definition “statistical” in nature, and human observers seem to overestimate object frequency compared to real-world measures^51^. We therefore compared the utility of the participant-rated measures of specificity and frequency we collected in Experiment 2 with measures derived from labelled image databases which should, at least in theory, be more objective estimates of object occurrence statistics of objects in the real world. This further allowed us to test the validity of subjective human ratings as estimators of object specificity and frequency.

We set up and compared five models predicting basic-level categorization accuracy using the scaled covariates object size on screen and eccentricity along with different measures of specificity and frequency (see Table 2). The first two models were based on the specificity and frequency ratings of our participants, either averaged across participants (M5) or non-averaged (trial-specific ratings, M6). The other three models used more objective measures of specificity and frequency that we calculated either from our hand-labelled stimulus set (M7), a subset of the ADE20K dataset only containing the 16 stimulus categories we used in our experiment (M8) or the full ADE20K dataset (M9). We then compared the fits of these models using the *AICcmodavg* package for R^52^.

**Table 2:** Comparison of models using different measures of object specificity and frequency to predict basic-level scene categorization accuracy in Experiment 2 and, as a validation, in Experiment 1.

Model	Experiment 2			Experiment 1		
	AICc	Δ AICc	Weight	AICc	Δ AICc	Weight
M5: Averaged ratings	13,377.61	0	1	11,591.78	0	1
M6: Human ratings	13,435.97	58.36	0			
M7: Own stimuli	13,454.36	76.75	0	11,790.18	198.40	0
M8: ADE subset	13,457.52	79.90	0	11,815.19	223.41	0
M9: ADE full database	13,482.72	105.11	0	11,841.17	249.39	0

Displayed are AICc values comparing non-nested models predicting basic-level scene categorization accuracy by different measures of object specificity and frequency and the standardized covariates object size and eccentricity.

The ratings used as predictors in models M5 and M6 were collected in Experiment 2, therefore model M6 (participant- and trial-specific ratings) cannot be computed for data from Experiment 1. See text for details.

Interestingly, the model based on averaged participant-rated measures of specificity and frequency (M5) provided the best model fit and yielded an AICc weight of 1, implying that, given our candidate set of models, the probability of this model being the best to predict new data is (close to) 1. The model containing the non-averaged participant-rated measures (M6) yielded the second-best model fit. The fact that this model fit worse than the averaged model implies that the mean of the ratings was more informative of scene categorization accuracy than the individual participant’s assessments, possibly because of outliers in the individual ratings that were mitigated in the averaged ratings (e.g. extreme values, misunderstanding of rating items by some participants, etc.). Remarkably, both models using rating-based measures fit the data better than the remaining three models that used more objective measures of specificity and frequency derived from database statistics. This indicates that human participants are apparently better in estimating which objects are informative of a scene’s category in a categorization task than real-world measures of object occurrence. And, interestingly, this advantage did not seem to be related to the number of distractor categories in the databases as human ratings outperformed all three database measures we calculated (M7–M9). Another surprising finding was that the model based on our database of labelled images (M7) was closely followed by the model based on a subset of the ADE20K database (M8), showing that the higher number of images in the subset of the ADE20K database (here 8,035 images in 16 different categories) did not out-perform the statistics we derived from labelling only 400 images. Lastly, the model calculating specificity and frequency measures from all categories of the ADE20K database (M9) yielded the worst model fit. While this model contained information from more than 22,000 scene images and 800 scene categories and therefore was more detailed, it was not able to capture the performance of our human participants, which seems to be more closely related to their subjective assumptions regarding object specificity and frequency (M5 & M6), or the objective statistics of specificity and frequency in the specific categories we used in the experiment (M7 & M8). The better performance of measures derived from databases only containing the 16 scene categories used in the experiment (M7 & M8) also supports the notion that participants adopted a task-specific mindset in which the presence of target objects in task-irrelevant distractor categories is not considered. This is further supported by higher correlations between the averaged ratings of specificity and frequency (M5) with the measures derived from our labelled stimuli (M7; 0.51 and 0.46 for specificity and frequency, respectively) compared to the correlation with the measures derived from the full ADE20K database (M9; 0.33 and 0.29, respectively; see Supplementary Fig. S3).

Lastly, we replicated our findings regarding the utility of different measures of specificity and frequency and assessed whether human ratings still outperform database measures when they are collected from an independent sample of participants—our human ratings might simply be a better predictor than database measures because they were collected from the same sample that they were later tested on. To account for this, we repeated the same model comparisons described above but this time predicted scene categorization performance of participants in Experiment 1 using averaged specificity and frequency ratings from the independent sample of participants in Experiment 2 (see Table 2, right column). We set up models identical to those specified above (except for model M6 that used participant- and trial-specific data) using scene categorization accuracy from Experiment 1 as the dependent variable. The results were the same: averaged human ratings were the best predictors of scene categorization accuracy, followed by statistics derived from our hand-labelled stimuli and the measures calculated based on the ADE20K database. These findings not only replicate the advantage of using human ratings, they also show that these ratings do not have to be participant-specific and seem to work similarly well when used on data of an independent sample of participants.

## Experiment 3

In Experiment 2, we identified the specificity of an object for a given scene category and its occurrence frequency as the object properties that were most diagnostic of human scene categorization. We further showed that these properties best predicted categorization performance when measured through averaged human ratings as compared to more objective statistics derived from databases of labelled images. However, one caveat in the interpretation of these findings is that the objects we presented varied in size and eccentricity which may have affected object recognizability and the relevance of certain object properties for scene categorization. This was also reflected in the large effect sizes of the object size and eccentricity covariates in the models predicting categorization accuracy (see Supplementary Tables S2 and S3). In Experiment 3, we therefore eliminated this factor by resizing all objects to the same size and presenting them at the fixation point (i.e. the centre of the image). Note that such a manipulation reduces ecological validity as objects rarely appear enlarged at our fovea during real-world scene viewing. However, the primary purpose of Experiment 3 was to replicate our findings from Experiment 2 while experimentally controlling for effects of object size and eccentricity to rule out that our findings are purely an effect of these covariates.

### Method

The method of Experiment 3 was identical to Experiment 2, except for the deviations noted below.

#### Participants

Fifty-two volunteers participated in Experiment 3. As in Experiment 2, we conducted separate power analyses for the nine object properties we tested here. We found that a sample size of 40 was sufficient to reach power > 0.80 for most object properties, but we decided to collect more data to account for potential removal of participants. We excluded data of two participants who did not complete both parts of the experiment and four participants who showed systematic response patterns in the categorization or rating task, leaving data of 46 participants for analysis (29 female, 16 male, 1 not specified; 18–39 years old, *M* = 24.5 years). None of the participants of Experiments 1 or 2 participated in Experiment 3.

#### Stimuli

We used the same subset of 240 base stimuli as described in Experiment 1, but we resized all cut-out objects so that they subtended a maximum of 256 pixels horizontally or vertically, depending on which object dimension was larger (see Fig. 1c). We then padded the objects with a 128-pixel, uniform grey margin so that the resulting stimuli had the same size as those in Experiments 1 and 2 (512 × 512 pixels).

#### Procedure

The procedure of Experiment 3 was identical to Experiment 2, except we discarded the diagnosticity rating item and presented the specificity and frequency rating items in the first part of the experiment whereas the remaining seven items were presented in random order in a separate part.

#### Analysis

Our analysis plan was mostly identical to that described in Experiment 2. We used data of 46 participants and discarded practice trials, leaving a total of 11,040 observations for analysis. In the Lasso model selection procedure and the subsequent comparisons between different measures of specificity and frequency, we did not include the covariates object size and eccentricity anymore since these were experimentally controlled for. After identifying usage frequency as an additional relevant predictor of scene categorization accuracy, we decided to run further analyses comparing models containing different measures of specificity and frequency both with and without usage frequency and report results of both analyses.

## Results and discussion

**Figure 4:**
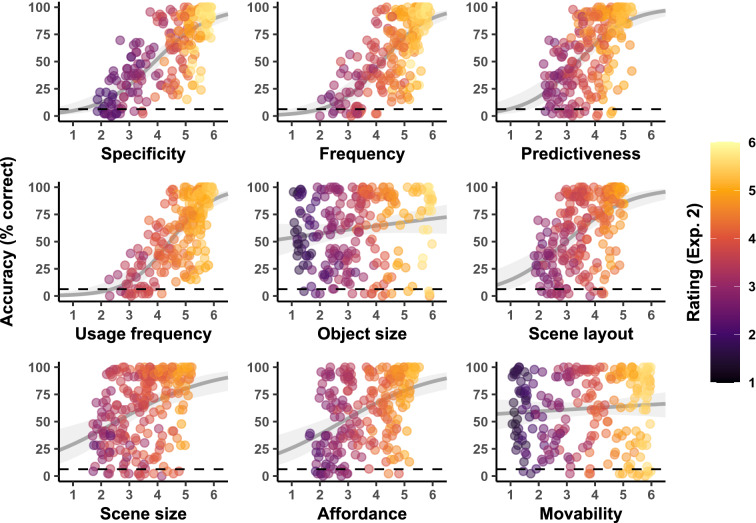
Observed basic-level scene categorization accuracy in Experiment 3 as a function of different ratings of object properties. Points represent the respective rating and accuracy averaged across participants for each individual image in Experiment 3. The points’ colours indicate the mean ratings of the same images and items in Experiment 2. Smoothed lines are fixed-effect binomial approximations for illustrative purposes only. The dashed lines indicate the expected chance level in the basic-level scene categorization task (6.25%).

The mean superordinate-level and basic-level categorization accuracy across images and participants were slightly higher compared to Experiment 2 (93.5% and 62.1%, respectively; indoor: 92.3% and 59.3%; outdoor: 94.6% and 65.0%). This implies that the resizing and centring may have helped recognizing some of the objects (presumably especially smaller objects, see Supplementary Figs. S4 and S5) and therefore also improved scene categorization. Figure 4 summarizes the observed basic-level categorization accuracy of each image in Experiment 3 as a function of the different ratings collected in this experiment. Additionally, the points’ colours indicate the averaged ratings collected in Experiment 2, showing that most ratings remained stable across tasks. Unsurprisingly, most affected by the object size and position manipulation were the ratings of object size (which was not correlated with the size of the object on the display anymore), scene size implication and scene layout implication. Note that the latter two rating items may have been rendered “undiagnostic” because changing the size and location of an object relative to the image frame may also have excluded important information to interpret the scene’s size and layout (e.g. a visually small table appearing distant from the observer will probably not be part of a kitchen but a restaurant scene). We therefore suggest treating results of these rating items with caution.

We repeated the Lasso variable selection procedure described above for data from Experiment 3 (see Supplementary Table S4). The optimal model once again contained the predictors specificity and frequency, but it also contained the predictor usage frequency (see Table 1) which, in contrast to the optimal model in Experiment 2, was a significant predictor of categorization accuracy, even though its effect was descriptively smaller compared to specificity and frequency. When running separate analyses for indoor and outdoor scenes, respectively, the optimal models once again contained specificity, frequency, and usage frequency for indoor scenes, but only specificity and frequency for outdoor scenes. In line with Experiment 2, it appears that specificity and frequency are the most diagnostic object properties, but indoor scene categorization accuracy was also predicted by additional, higher-level information: the frequency of our interaction with the object. This effect appears to be mostly driven by an increased categorization performance for smaller objects in indoor scenes after resizing (see Supplementary Fig. S5). On the other hand, outdoor scenes showed a lower average change in categorization accuracy after resizing (indoor scenes: 14.2 percentage points, outdoor scenes: 4.8 percentage points) and a more heterogeneous relationship between usage frequency and accuracy change (see Supplementary Fig. S5). We therefore conclude that the emergence of usage frequency as a relevant predictor of scene categorization accuracy in Experiment 3 is mostly related to better recognition of smaller objects after resizing that are frequently used in indoor scenes. Note that, while this implies that smaller objects can also be diagnostic when they are correctly recognized, viewing conditions during natural scene categorization usually do not allow close-up inspection of smaller objects and are rather characterized by a glimpse from some distance.

The comparison of different measurements of specificity and frequency (see Supplementary Table S5) once again revealed better fits for models containing human ratings (trial-specific or averaged) compared to models containing database-derived measures (our own labelled stimuli and the ADE20K statistics). However, in contrast to Experiment 2, the trial-specific ratings yielded better fits than the averaged human ratings in this experiment. This may be related to the overall higher difficulty of recognizing smaller objects in Experiment 2. Some participants may not have recognized smaller objects within the 50-ms presentation time, but they may have given high specificity and frequency ratings after the experiment when stimuli were presented until response, thus leading to poor predictions of the ratings. In Experiment 3, on the other hand, smaller objects were easier to recognize due to the standardized size. The trial-specific ratings may therefore be better predictors than the average scores in this case because they contain more information. A second noteworthy difference in this analysis is that model M8 (ADE20K subset) fit the data better than model M7 (based on our labelled stimuli). While the difference between these models was already small in Experiment 2, resizing and centring of the objects apparently lead to better predictions of the specificity and frequency measures based on real-world data. This may once again be related to improved recognition of smaller objects in Experiment 3, the specificity and frequency of which may be better measured based on the bigger, more accurate statistics derived from the ADE20K database.

## Experiment 4

After showing that object specificity and frequency are the most relevant predictors of human scene categorization accuracy and demonstrating that they are best measured using human ratings in Experiments 2 and 3, we were last interested in the role that scene-specific visual information (the specific appearance of, for example, a chair from a restaurant vs. a chair from a classroom) plays during scene categorization in comparison to conceptual object information (merely understanding that a presented object is a chair). We therefore replaced the objects we had cut out from real-world scene images with prototypical object images not conveying scene-specific visual information in Experiment 4 while keeping the same experimental paradigm and analysis strategy as in Experiments 2 and 3. Once again, this manipulation further reduced the ecological validity of the stimuli, but in comparing the results of this experiment to our previous findings, we could assess the role of scene-specific vs. conceptual object information during human scene categorization. Scene-specific visual information may be crucial for the assessment of object properties such as specificity and spatial layout and therefore change the diagnostic object properties we identify using the Lasso procedure. However, if object diagnosticity is primarily driven by conceptual object information, we would not expect the relevant predictors for human scene categorization to change after controlling for scene-specific visual information. Additionally, controlling for the effect of scene-specific visual information may also affect the predictiveness of human ratings and database measures of object specificity and frequency. One possible explanation for the higher predictive power of human ratings in Experiments 2 and 3 could be that humans can visually tell apart chairs from a restaurant versus a classroom whereas the labels generated for the database do not coney this information (for the database both objects are simply instances of “chairs”). If this were the case, we would expect equal predictiveness of human ratings and database measures of specificity and frequency using prototypical object stimuli.

### Method

The method of Experiment 4 was identical to Experiment 3, except for the deviations noted below.

#### Participants

Fifty-three volunteers participated in Experiment 4. In line with previous experiments, separate power analyses for the nine object properties we tested here showed that a sample size of 40 was sufficient to reach power > 0.80 for most object properties. We once again collected more data to account for potential removal of data. We excluded data of nine participants who did not complete both parts of the experiment and one participant who showed a systematic response pattern in the categorization task, leaving data of 43 participants for analysis (15 female, 28 male; 18–40 years old, *M* = 25.7 years). None of the participants of Experiments 1, 2 or 3 participated in Experiment 4.

#### Stimuli

We used 129 prototypical images of the objects presented in the previous experiments as stimuli in Experiment 4 (see Fig. 1d). We first identified the unique objects presented in each scene category and then searched for a prototypical object image for each category in a web search (e.g. one chair for the restaurant category, another chair for the classroom category, etc.). Criteria for inclusion were that the displayed object was a typical exemplar of the respective object category, photographed from an angle that allowed easy recognition (i.e. no frontal or top view), and presented centrally on white background without any other objects or scene information. We then resized all objects to fill a 512 × 512 pixel image in their largest dimension.

#### Analysis

Our analysis plan was identical to that described in Experiment 3. We used data of 43 participants and discarded practice trials, leaving a total of 5547 observations for analysis.

## Results and discussion

**Figure 5:**
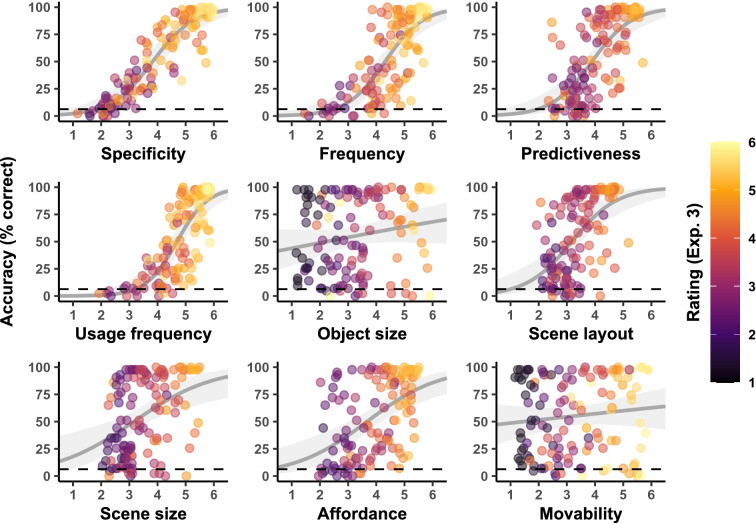
Observed basic-level scene categorization accuracy in Experiment 4 as a function of different ratings of object properties. Points represent the respective rating and accuracy averaged across participants for each individual image in Experiment 4. The points’ colours indicate the mean ratings for images showing the same object in Experiment 3. Smoothed lines are fixed-effect binomial approximations for illustrative purposes only. The dashed lines indicate the expected chance level in the basic-level scene categorization task (6.25%).

The mean superordinate-level and basic-level categorization accuracy across images and participants were descriptively lower compared to Experiment 3 (93.3% and 55.2%, respectively; indoor: 95.5% and 54.4%; outdoor: 90.4% and 56.2%), implying that the removal of scene-specific visual information in Experiment 4 did impact the diagnosticity of single objects for human scene categorization but did not render objects completely undiagnostic. Figure 5 summarizes the observed basic-level categorization accuracy of each image in Experiment 4 as a function of the different ratings collected in this experiment. Additionally, the points’ colours indicate the averaged ratings collected for stimuli displaying the same object in the respective category in Experiment 3. Note that the ratings of Experiment 3 and 4 are not as strongly correlated as ratings of Experiment 2 and 3 (compare Fig. 4), indicating that the removal of scene-specific visual information in Experiment 4 also led to different evaluations of object properties.

We repeated the Lasso variable selection procedure described above for data from Experiment 4 (see Supplementary Table S6). Replicating our findings of Experiment 3, the optimal model contained the predictors specificity, frequency, and usage frequency, indicating that object diagnosticity for scene categorization is primarily based on conceptual and not scene-specific visual information conveyed by objects. Additionally, we once more found that usage frequency is a relevant predictor of scene categorization when objects are easily recognized (i.e. when they are resized, centred and here also prototypical), even though these viewing conditions are not typical for real-world scene perception. A separate analysis for indoor scenes additionally revealed action affordance (significant) and predictiveness (non-significant) as relevant predictors. For outdoor scenes, only specificity and usage frequency were contained in the optimal model. Note, however, that the separate analyses for indoor and especially outdoor scenes are based on less trials which makes the results of the Lasso procedure less stable. We therefore suggest interpreting these results with caution. Lastly, we once again compared the capacity of different measurements of specificity and frequency to predict scene categorization accuracy (see Supplementary Table S7). In line with Experiments 2 and 3, we found better fits for models based on human ratings (trial-specific or averaged) compared to models based on database-derived measures (our own labelled stimuli and the ADE20K statistics). However, the ranking of these models differed slightly. When we included usage frequency as a predictor, the model based on statistics derived from our own labelled stimuli (M7) yielded the worst fit to the data. This may suggest that excluding scene-specific visual object information in Experiment 4 while controlling for usage frequency (which is not represented in the databases) was successful in creating comparable conditions in which the ADE20K database outperformed our smaller database. However, the difference between models M7 and M9 was very small (Δ AICc = 0.36), and human ratings were still better measures of specificity and frequency in this experiment, demonstrating that the advantage of human ratings over database measures was not purely an effect of scene-specific visual information conveyed by the objects that was only available to human raters.

## General discussion

In this study, we put object-centred theories of human scene categorization to a test, which claim that scenes can be categorized through mere recognition of single objects^7,8,23^. We presented scene stimuli reduced to single, cut-out objects in a scene categorization task and assessed the precise properties that made these objects diagnostic for scene categorization. Experiment 1 showed that participants can still categorize scenes reduced to single objects. Furthermore, participant-rated object diagnosticity proved to be a good predictor of categorization accuracy, implying that we have an intuitive understanding of objects that are indicative of a scene’s category, whereas anchorness showed a weaker relation and did not contribute information beyond that already contained in the diagnosticity ratings. We therefore conclude that anchor objects are usually diagnostic, but anchorness itself is not necessarily a property of diagnostic objects. In Experiment 2, we showed that scene category information can be extracted within 50 ms of object presentation while also assessing the precise properties that make an object diagnostic and how these are best measured. We identified object specificity and frequency as the most important object properties predicting human scene categorization, and we showed that these can be well approximated using human ratings. In comparison, more objective, database-derived measures of specificity and frequency were worse predictors of categorization accuracy, indicating that human scene categorization behaviour is better explained by human intuition than real-world statistics of object occurrence. In Experiments 3 and 4, we replicated our findings from Experiment 2 while experimentally controlling for the influence of object size, eccentricity, and scene-specific object information. We once again showed that specificity and frequency are the most important object properties for human scene categorization, but we also found a significant effect of object usage frequency which we primarily attributed to better recognition of smaller objects after resizing them in Experiments 3 and 4. In summary, this study shows that single objects are useful and often sufficient for human scene categorization when they frequently occur in the target scene category and rarely in other task-relevant distractor categories.

### Object recognition as one plausible route towards scene categorization

On a theoretical level, our findings support object-centred theories of human scene categorization^7,8^. We present evidence for the sufficiency of single objects to indicate the category of the scene they were taken from. In Experiment 1, using a rather long presentation time of 1000 ms, 73% of trials were correctly categorized at the basic level. Even after drastically reducing presentation time to 50 ms in Experiment 2, 53% of trials were still correctly categorized (62% after resizing and centring of objects in Experiment 3 and 54% after removal of scene-specific visual information in Experiment 4). This shows that object information is not only useful, it can also be extracted from stimuli quickly, which further supports the first route suggested by Biederman^7^ that is thought to give rise to an initial scene representation by quickly conveying information about one (or several) of the prominent objects in the scene. Importantly, our experiments show that scene categorization does not necessarily require multiple objects^23,53,54^ or explicit information regarding object co-occurrence^55^. Instead, we demonstrate that a single object can be sufficient for scene categorization. Note that, while this finding may sound trivial, it is important to establish the diagnosticity of the objects we used here first to then assess the precise object properties that make an object diagnostic (see below).

Despite the high average utility of objects in our experiments, there was also a significant proportion of objects in all four experiments that were not suited to identify the underlying scene category. This implies that not all objects are sufficient, and the object-centred approach is only one possible route to human scene categorization. When the presented object is unambiguously related to a scene category (i.e. it is specific to that category and frequently occurs in it), the “object route” may deliver a fast and reliable solution to the categorization problem. However, in other cases, this process may come to an incorrect solution (e.g. when there is a shower in a kitchen), it might be insufficient for an unambiguous categorization (e.g. a tap might occur in both a kitchen or a bathroom), or other information (e.g. spatial layout information, global scene properties) might be used in conjunction with or independently of object information (e.g. in scenes that contain few objects such as deserts). Nonetheless, object information usually seems to be a useful source of information, yet it is unlikely to be the sole candidate driving human scene categorization.

The fact that a single object is sufficient to allow for scene category access is in line with studies showing facilitating or interfering effects of semantically consistent or inconsistent foreground objects on scene categorization^11,24,25,56^. Note, however, that the information and mechanisms that dictate how an inconsistent object interferes with scene categorization must not necessarily be the same by which such an object gives rise to another scene category^54,57^. Our results are therefore relevant as they explicitly show that a single object can allow for direct scene category access and not only interfere with the understanding of another scene the object was pasted on.

### Diagnostic object properties for human scene categorization

A second important finding of our study is that human scene categorization behaviour based on single objects is best explained by the objects’ specificity for the given scene category and the frequency of the object within that category. This implies a rather “statistical” view of object diagnosticity: Objects appear to be diagnostic when they often occur in the target scene category and rarely in other task-relevant distractor categories. A stove, for example, is very diagnostic for the kitchen category because almost all kitchens contain a stove (frequency), and stoves rarely occur in other categories (specificity). Objects should be less diagnostic if only one of the two criteria is met: This includes objects that frequently occur in a scene category but also in other scenes (e.g. books in living rooms) or objects that are specific to a scene category but do not frequently occur in it (e.g. a rice cooker in kitchen scenes). This finding is generally in line with proposals claiming that objects are diagnostic when they have a high occurrence probability and a strong thematic relation to a scene^8,23,31^ and definitions of diagnosticity based on object co-occurrence statistics^30,55^.

Note that usage frequency also emerged as a relevant predictor after resizing and centring of objects in Experiments 3 and 4 but not in Experiment 2. However, we maintain that presenting objects in their original size and location is the appropriate approach to assess relevant information for human scene categorization, because it more closely resembles the viewing conditions under which we usually categorize real-world scenes. We rarely find ourselves standing in the middle of a scene, suddenly having to categorize it. Categorization usually occurs when we are visually exposed to a scene for the first time (which typically is at larger distances, thus reducing the visual angle at which the objects in a scene are perceived) and not after we have moved through a scene, navigated towards smaller objects, and looked at them close-up.

Our data also partly suggest that objects holding predictions about the presence of other objects are diagnostic^7,32^. However, this relationship was weak and only seemed to matter for indoor stimuli, probably because of their functional and more predictable design^33^. Other properties of anchor objects (size and movability) also only appeared in models for indoor scenes, and had, if at all, very little predictive power. The claim that more discriminable objects are diagnostic^7^ is supported by our data. Object size and, to a lesser extent, eccentricity explained significant proportions of variance in categorization accuracy, and objects that were less recognizable led to lower categorization accuracy (see Supplementary Fig. S2). After resizing and centring objects in Experiments 3, we observed an increase in both superordinate-level and basic-level categorization accuracy by 6.6 and 9.5 percentage points, respectively, which further supports the idea that the first object that meets the eye may already be able to activate plausible predictions of the scene category.

Interestingly, two popular claims regarding relevant higher-level and lower-level information for scene categorization were not supported by our data: First, in contrast to scene-centred theories of scene categorization highlighting the role of global scene information^9,10^, object diagnosticity was not strongly related to the quality of single objects to convey information about scene size and spatial layout. However, this should not be seen as evidence against these theories because spatial layout usually emerges from the arrangement of several objects^10^, and scenes rarely consist of only one layout-defining object as in our stimuli. Second, action affordances elicited by objects were not strong predictors of scene categorization performance either, even though scenes have previously been proposed to be categorized by function^36,37,58^. This might indicate that single objects are not sufficient to explain the more complex affordance character elicited by a complete scene. After all, there are several objects in different scenes that afford, for example, sitting, but the presence of other objects that afford cooking or working will tell the observer if they are looking at a kitchen or an office. And even within a scene different “phrases” (sub-groups of objects clustering around an anchor object) serve different functions^33^. For example, bathroom scenes usually contain a sink phrase that affords washing hands, brushing teeth, and combing hair whereas the shower and toilet phrase afford different actions. Furthermore, scene functions may only be relevant for more detailed, subordinate-level categorization^4^ and may therefore not have contributed to the categorization of the 16 rather well distinguishable basic-level scene categories we used here.

On the other hand, the emergence of usage frequency in the originally optimal model in Experiment 2 and the replications in Experiments 3 and 4 underlines the relevance of our interactions with objects, even though usage frequency itself was not as strongly related to categorization performance as specificity and frequency. For scene categorization, it therefore does not seem to matter to understand the action possibilities an object affords, it rather seems to be important how often we use (or have used) it in a scene. This may also hint at another possible advantage of human ratings over more objective, data-driven measures of object properties (see below): we have *interacted* with objects countless times, a database of images has not. Previous research suggests that interacting with task-relevant objects increases our memory for their position^59^. Our findings could be interpreted as suggesting that these memories are long-lasting and also relevant for later scene categorization.

It is further important to note that other object properties besides specificity and frequency appeared to be primarily relevant for indoor scenes. In both Experiments 2 and 3, the Lasso procedure run on outdoor scenes consistently revealed only specificity and frequency as diagnostic predictors. Indoor scene categorization, on the other hand, was also predicted by predictiveness, movability and object size (three properties of anchor objects)^32^ in Experiment 2 and usage frequency in Experiment 3. While the predictive power of all these variables was rather low, and only usage frequency in Experiment 3 reached statistical significance, this observation implies that different types of information seem to matter for different scene categories. In the case of indoor scenes, this entails higher-level properties such as predictiveness of other objects and usage frequency. We believe that this is in part due to the functional design of indoor scenes that are (at least in the case of the eight categories we used here) designed to carry out certain actions and contain objects in predictable locations to be used and interacted with^33^. In comparison, these properties may be less relevant for scene categories that afford fewer actions and do not follow strict scene grammar rules (e.g. a forest or a mountain scene). Note that the categorization accuracy was also slightly lower for indoor compared to outdoor scenes across experiments which may be related to an overall bias to respond with the outdoor category at short presentation times^20,50^.

### Measuring diagnostic object properties

More generally, we believe that the predominance of object specificity and frequency implies that participants in our categorization experiments adopted a task-specific mindset in which they mainly focussed on object co-occurrence statistics that were best suited to maximize discriminability between task-relevant scene categories^60^. For the specific task, stimuli, and scene categories we used here, this seemed to entail high-frequency objects with little overlap across categories. And while specificity and frequency are by definition “statistical” in nature, they seem to best predict categorization behaviour when they are measured through human ratings and not using database-derived measures. This is somewhat surprising as one could argue that measures derived from databases containing thousands of images are more objective approximations of object occurrence statistics in the real world than human ratings. Furthermore, humans tend to overestimate object frequency^51^. However, the database-derived measured we used here were not as predictive of categorization behaviour as the perceived utility of objects in discriminating the specific categories used in the task, possibly due to database biases (e.g. centre bias, little clutter in stylized scene images, objects hidden inside furniture, etc.) or differences between the real-world scene experiences of the German undergraduate students tested here and the ADE20K database (which contains a lot of images representing typical American environments). The subjective nature of the perceived diagnosticity of objects is further demonstrated by higher predictive power (in most experiments) of the specificity measure based on only the 16 categories we used in the experiment compared to the full ADE20K database, which implies that our participants did not necessarily consider the occurrence of objects in other, task-irrelevant categories. When we enter someone’s private apartment and look into a room with a set table and chairs, we are probably sure that this must be the dining room and do not consider that these objects could occur in a restaurant scene as well^61,62^.

On the other hand, compared to databases, human raters also have a qualitatively different understanding of the scenes they have experienced. First, they have moved through them, interacted with them, have even designed some of them, and created 3D maps of them allowing for predictions which objects are most likely present outside the current field of view^63^ or where hidden objects are^64^. Once again, this is also in part reflected by the emergence of usage frequency and predictiveness of other objects as additional predictors in Experiments 3 and 4. Second, Experiments 3 and 4 underline that humans can visually and conceptually distinguish between chairs typically found in a kitchen, a classroom, or an office. We can therefore say that one chair is highly specific to kitchens whereas another chair is highly specific to classrooms or offices. In contrast, the database measures we used here operate on text labels and neither have the visual information nor the conceptual knowledge to make these distinctions, and all instances of chairs are therefore simply chairs that are not specific to either category. However, we still found an advantage of human ratings after replacing the cut-out objects with prototypical object images in Experiment 4, showing that scene-specific visual information is not the sole cause of this advantage and instead conceptual knowledge seems to play a rather important role.

Note that the advantage of human ratings over database statistics persisted when we predicted categorization accuracy from Experiment 1 using ratings collected in Experiment 2, indicating that this is not merely an effect of testing our models on the same participants and data we derived them from. This suggests that human ratings may be a cost-efficient and suitable (in some cases even preferrable) alternative to database-derived object statistics, especially in cases when no statistics are available for specific objects or stimulus sets. However, we must point out that all our experiments were run on the same base stimuli and scene categories, and the relevant object properties and best ways to measure them may vary as a function of these parameters (see for example the discussion of object occurrences in task-irrelevant distractor categories above). Furthermore, we used objects as stimuli that were usually well visible in the scenes and could be cut-out so that they were still recognizable in isolation. This excludes a lot of smaller objects, partly occluded objects, objects photographed from an unusual or non-canonical angle, and objects that are difficult to recognize in a 2-D photograph (e.g. a piece of paper or a document). The resulting stimulus database we used here is therefore—like so many—biased and may not account for natural viewing conditions. Nonetheless, we demonstrate the utility of object information for scene categorization across a set of diverse scene categories (private and public indoor scenes, natural and man-made outdoor scenes) and the reliability of human ratings as predictors of scene categorization behaviour.

## Conclusions

In summary, our study provides evidence in favour of object-centred theories of human scene categorization, showing that single objects are useful and often sufficient for human scene categorization. However, not all objects are equally diagnostic for scene categorization, and the “object route” to categorization may fail. Among the objects properties we assessed, specificity and frequency were the best predictors of categorization performance, indicating that objects that frequently occur in a given scene category and rarely in other task-relevant distractor categories are diagnostic. Interestingly, human ratings of specificity and frequency predicted categorization performance better than more objective, real-world object occurrence statistics derived from labelled image databases. This indicates that participants attune themselves to the categorization task and the specific scene categories used and flexibly make use of the information that is most relevant to their current task goals.

## Supplementary Information

Supplementary Information.

## Data Availability

All reported data, analysis code and supplementary materials are publicly available on OSF: https://osf.io/rhq3k/. We have no known conflict of interest to disclose.
