# Supplementary material for: Disentangling diagnostic object properties for human scene categorization
Source: Sci Rep. 2023 Apr 11;13:5912. doi: 10.1038/s41598-023-32385-y (PMC10090043; doi:10.1038/s41598-023-32385-y)
Supplement: Supplementary file 1 — Supplementary Information. [file 41598_2023_32385_MOESM1_ESM.pdf]

Supplementary information for

Disentangling Diagnostic Object Properties for Human Scene Categorization

Sandro L. Wiesmann[1*] and Melissa L.-H. Võ[1]

[1]Department of Psychology, Johann Wolfgang Goethe-Universität, Frankfurt, Germany

*Correspondence concerning this article should be addressed to Sandro Luca Wiesmann, Department of Psychology, Goethe University Frankfurt, Theodor-W.-Adorno-Platz 6, 60323 Frankfurt am Main, Germany. Email: wiesmann@psych.uni-frankfurt.de

Experiment 1: Instructions for diagnosticity and anchorness ratings

The following instructions (original in German) were presented before the rating blocks and, as a reminder, at the beginning of each block in Experiment 1:

First, please indicate how **diagnostic** the presented object is for the indicated scene category. Diagnostic here means **how unambiguously one can infer the indicated scene category from the presented object**. A beach chair, for example, is very diagnostic for the category *beach* because beach chairs usually only occur in beach scenes. One can therefore normally “make the diagnosis” *beach* based on the object *beach chair*. In contrast, a towel is not very diagnostic for the category *beach* because towels also occur, for example, in bathroom and swimming pool scenes.

Second, please indicate whether the presented object is an **anchor object** for other objects. Anchor objects are objects that can **provide information regarding the presence and position of other related objects**. Anchor objects are **usually large and stationary** whereas the related objects are usually small and movable. For example, a sink is an anchor object for the soap because the soap can usually be found on top of the sink. Similarly, a shower is an anchor object for shampoo because shampoo can usually be found inside the shower. On the other hand, a football is not an anchor object because it is movable and does not provide reliable information regarding the presence and position of other objects around it. Neither is a fork, which usually occurs together with a knife, but which is easily movable in space.

Supplementary Figure S1

Observed superordinate-level scene categorization accuracy in Experiment 2 as a function of different ratings of object properties.

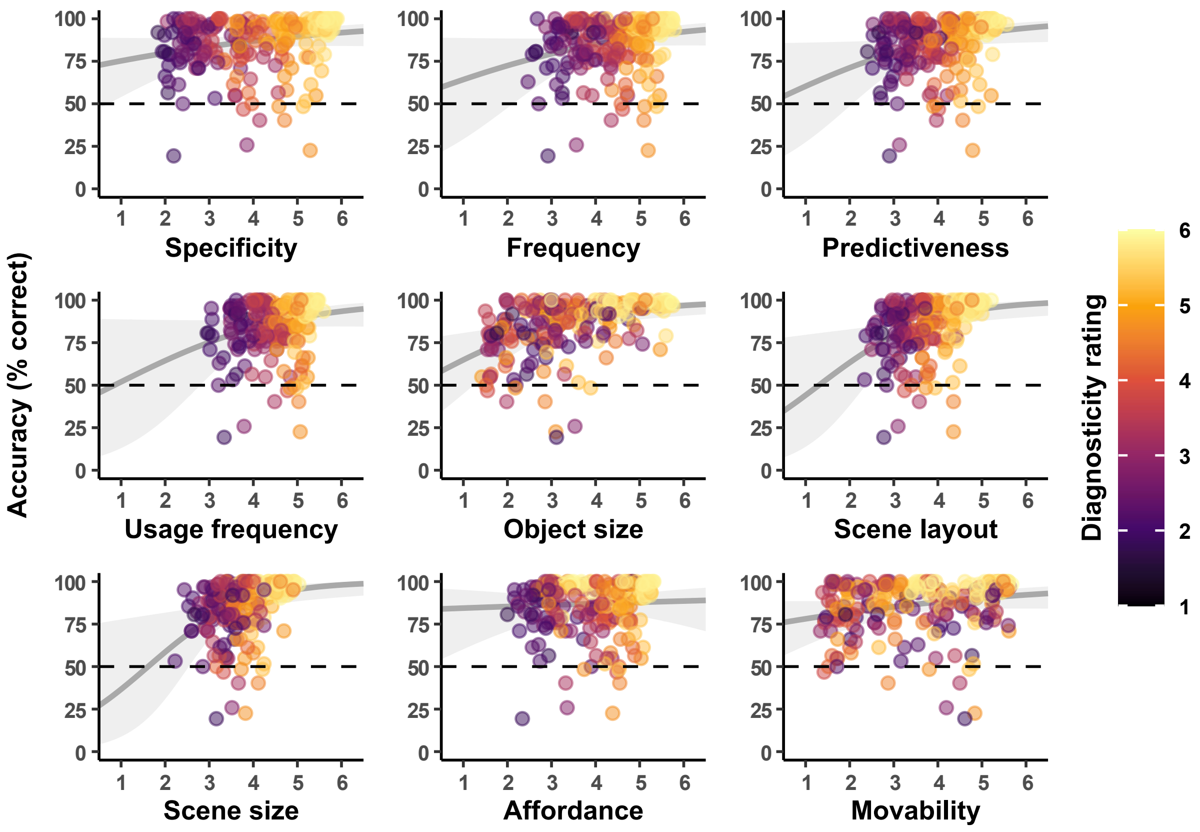

Note. Points represent the respective rating and accuracy averaged across participants for each individual image. Smoothed lines are fixed-effect binomial approximations for illustrative purposes only. The dashed lines indicate the expected chance level in the superordinate-level scene categorization task (50%).

Supplementary Figure S2

Observed (a) superordinate-level and (b) basic-level scene categorization accuracy in Experiment 2 as a function of object size on screen and object recognition.

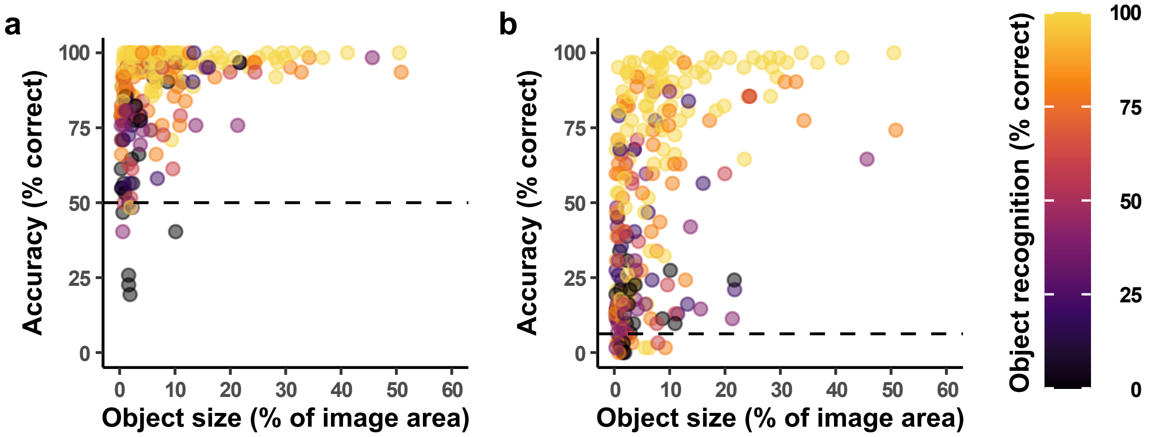

Note. Points represent data averaged across participants for each individual image. The dashed lines indicate the expected chance level in the superordinate-level (50%) and basic-level scene categorization task (6.25%).

Supplementary Figure S3

Correlations between different measures of object specificity and frequency in Experiment 2.

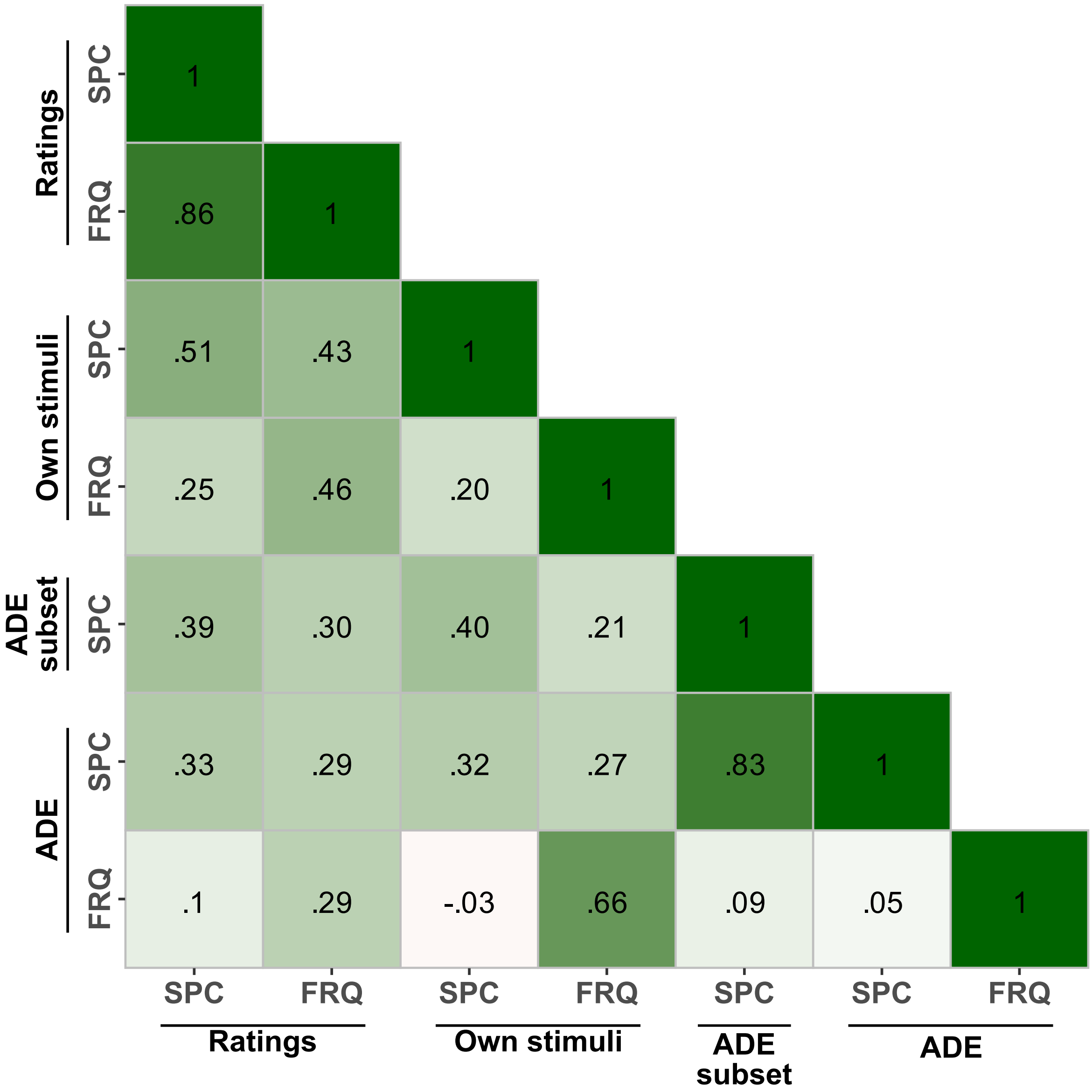

Note. SPC = Specificity, FRQ = Frequency, ADE = ADE20K dataset, ADE subset = ADE reduced to the 16 categories (and their synonyms) used in the experiment. Frequency measures for ADE subset and ADE are identical. See text for details.

Supplementary Figure S4

Observed basic-level scene categorization accuracy in Experiment 2 and 3 as a function of (a) object size on screen and (b) eccentricity.

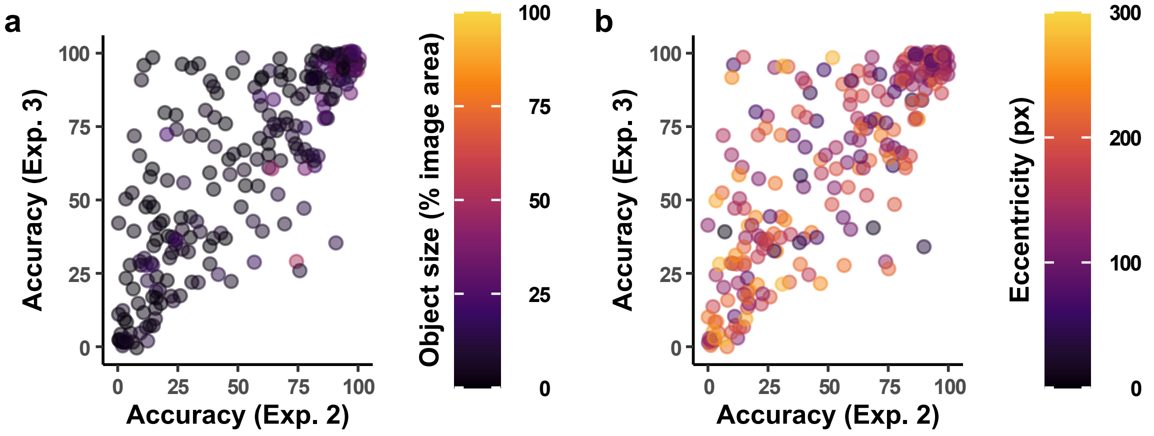

Note. Points represent mean accuracy (% correct) across participants for each individual image.

Supplementary Figure S5

Observed change in the usage rating and basic-level scene categorization accuracy from Experiment 2 to 3 as a function of the object size on screen (before resizing in Experiment 3) for (a) indoor and (b) outdoor scenes.

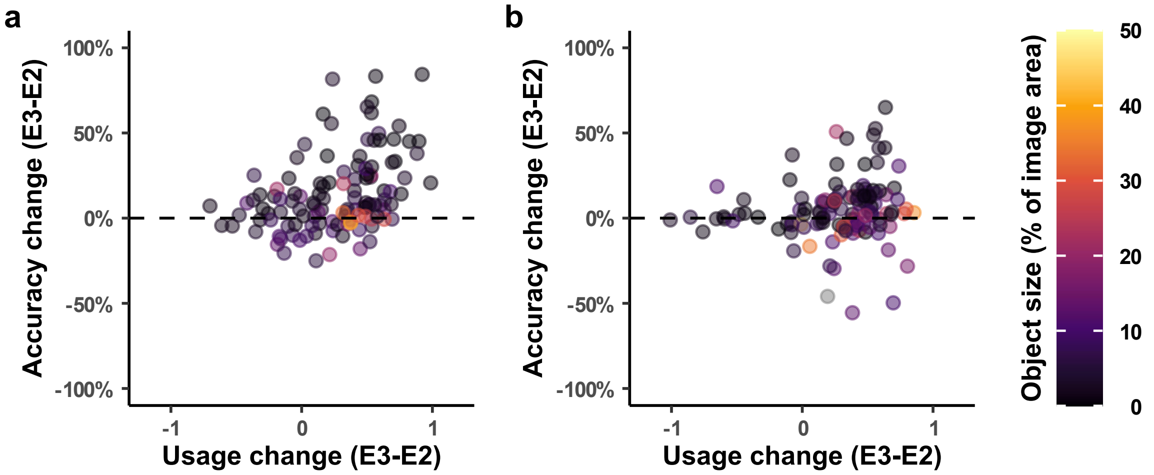

Note. Points represent data averaged across participants for each individual image. E2 = Experiment 2, E3 = Experiment 3.

Supplementary Table S1

Results of models predicting scene-categorization accuracy in Experiment 1.

	Superordinate-level categorization				Basic-level categorization			
Predictors	Logit	CI	*z*	*p*	Logit	CI	*z*	*p*
Model M1								
(Intercept)	3.98	3.69 – 4.27	27.05	<.001	1.60	1.27 – 1.94	9.42	<.001
Object size	0.40	0.16 – 0.64	3.30	.001	0.66	0.37 – 0.95	4.45	<.001
Eccentricity	-0.05	-0.21 – 0.11	-0.62	.534	-0.41	-0.69 – -0.12	-2.83	.005
Model M2								
(Intercept)	3.21	2.83 – 3.60	16.49	<.001	-0.46	-0.89 – -0.04	-2.13	.033
Object size	0.29	0.05 – 0.52	2.39	.017	0.43	0.18 – 0.67	3.45	.001
Eccentricity	0.01	-0.14 – 0.17	0.17	.867	-0.27	-0.51 – -0.04	-2.28	.023
Diagnosticity	0.18	0.11 – 0.25	5.08	<.001	0.47	0.40 – 0.55	12.51	<.001
Model M3								
(Intercept)	3.69	3.33 – 4.06	19.99	<.001	1.04	0.65 – 1.44	5.13	<.001
Object size	0.34	0.10 – 0.58	2.81	.005	0.56	0.30 – 0.82	4.22	<.001
Eccentricity	-0.03	-0.19 – 0.12	-0.42	.674	-0.37	-0.64 – -0.11	-2.75	.006
Anchorness	0.08	0.02 – 0.15	2.40	.016	0.16	0.10 – 0.23	5.21	<.001
Model M4								
(Intercept)	3.21	2.81 – 3.61	15.67	<.001	-0.53	-0.97 – -0.08	-2.32	.020
Object size	0.29	0.05 – 0.53	2.35	.019	0.40	0.17 – 0.62	3.45	.001
Eccentricity	0.02	-0.14 – 0.17	0.22	.829	-0.27	-0.51 – -0.03	-2.25	.025
Diagnosticity	0.19	0.11 – 0.28	4.51	<.001	0.48	0.40 – 0.55	12.67	<.001
Anchorness	-0.01	-0.10 – 0.08	-0.25	.806	0.01	-0.05 – 0.06	0.26	.798

Note. The covariates object size and eccentricity were standardized.

Supplementary Table S2

Results of models derived from the Lasso procedure for basic-level scene categorization accuracy in Experiment 2.

	Optimal model				Sparse model			
Predictors	Logit	CI	*z*	*p*	Logit	CI	*z*	*p*
(Intercept)	-0.78	-1.25 – -0.31	-3.28	.001	-0.62	-1.04 – -0.20	-2.86	.004
Object size	1.07	0.71 – 1.42	5.84	<.001	1.06	0.70 – 1.43	5.72	<.001
Eccentricity	-0.52	-0.76 – -0.27	-4.13	<.001	-0.51	-0.75 – -0.28	-4.29	<.001
Specificity	0.11	0.06 – 0.15	4.47	<.001	0.11	0.07 – 0.15	5.28	<.001
Frequency	0.08	0.04 – 0.12	3.57	<.001	0.09	0.05 – 0.13	4.29	<.001
Predictiveness	0.04	-0.01 – 0.09	1.73	.084				
Usage frequency	0.01	-0.04 – 0.07	0.40	.688				

Note. Displayed are fixed effects of the full models including random slopes for all predictors. The covariates object size and eccentricity were standardized. See text for details.

Supplementary Table S3

Results of the optimal models derived from the Lasso procedure predicting basic-level scene categorization accuracy in Experiment 2 for subsets of indoor and outdoor scenes.

	Indoor scenes				Outdoor scenes			
Predictors	Logit	CI	*z*	*p*	Logit	CI	*z*	*p*
(Intercept)	-1.07	-1.77 – -0.37	-3.00	.003	-0.27	-0.85 – 0.31	-0.92	.355
Object size	1.00	0.60 – 1.40	4.94	<.001	1.13	0.47 – 1.79	3.37	.001
Eccentricity	-0.40	-0.72 – -0.08	-2.45	.014	-0.66	-1.03 – -0.29	-3.48	.001
Specificity	0.10	0.04 – 0.16	3.03	.002	0.12	0.05 – 0.20	3.40	.001
Frequency	0.08	0.02 – 0.14	2.79	.005	0.09	0.03 – 0.15	2.75	.006
Predictiveness	0.04	-0.04 – 0.12	0.98	.327				
Movability	0.00	-0.05 – 0.05	-0.03	.974				
Estimated object size	0.01	-0.06 – 0.08	0.24	.807				

Note. Displayed are fixed effects of the full models including random slopes for all predictors. The covariates object size and eccentricity were standardized. Movability was inverted (higher values indicating stationary objects).

Supplementary Table S4

Results of the optimal models derived from the Lasso procedure for basic-level scene categorization accuracy in Experiment 3.

	All scenes				Indoor scenes				Outdoor scenes			
Predictors	Logit	CI	*z*	*p*	Logit	CI	*z*	*p*	Logit	CI	*z*	*p*
(Intercept)	-1.95	-2.48 – -1.42	-7.18	<.001	-1.73	-2.51 – -0.94	-4.30	<.001	-1.70	-2.41 – -0.98	-4.64	<.001
Specificity	0.31	0.24 – 0.38	8.58	<.001	0.30	0.22 – 0.38	7.26	<.001	0.31	0.22 – 0.41	6.44	<.001
Frequency	0.19	0.13 – 0.25	6.21	<.001	0.14	0.08 – 0.21	4.38	<.001	0.26	0.17 – 0.35	5.70	<.001
Usage frequency	0.12	0.05 – 0.18	3.54	<.001	0.11	0.03 – 0.19	2.86	.004				

Note. Displayed are fixed effects of the full models including random slopes for all predictors.

Supplementary Table S5

Comparison of models using different measures of object specificity and frequency to predict basic-level scene categorization accuracy in Experiment 3, either with usage frequency as an additional predictor or without.

	With usage frequency			Without usage frequency		
Model	AICc	Δ AICc	Weight	AICc	Δ AICc	Weight
M6: Human ratings	9408.13	0	1	9436.53	0	1
M5: Averaged ratings	9544.74	136.61	0	9617.77	181.24	0
M8: ADE subset	9663.37	255.25	0	9769.64	333.10	0
M7: Own stimuli	9679.86	271.73	0	9776.43	339.89	0
M9: ADE full database	9691.96	283.83	0	9799.04	362.51	0

Note. Displayed are AICc values comparing non-nested models predicting basic-level scene categorization accuracy by different measures of object specificity and frequency (and usage frequency in the left models). See text for details.

Supplementary Table S6

Results of the optimal models identified by the Lasso procedure for basic-level scene categorization accuracy in Experiment 4.

	All scenes				Indoor scenes				Outdoor scenes			
Predictors	Logit	CI	*z*	*p*	Logit	CI	*z*	*p*	Logit	CI	*z*	*p*
(Intercept)	-3.48	-4.24 – -2.72	-8.98	<.001	-3.86	-4.94 – -2.78	-7.02	<.001	-2.76	-3.87 – -1.66	-4.90	<.001
Specificity	0.56	0.45 – 0.67	10.31	<.001	0.57	0.44 – 0.69	9.07	<.001	0.51	0.37 – 0.65	7.31	<.001
Frequency	0.18	0.09 – 0.27	3.81	<.001	0.19	0.09 – 0.29	3.67	<.001				
Usage frequency	0.17	0.08 – 0.25	3.64	<.001	0.14	0.01 – 0.28	2.03	.042	0.24	0.12 – 0.36	4.03	<.001
Affordance					0.12	0.02 – 0.22	2.34	.019				
Predictiveness					-0.02	-0.15 – 0.11	-0.25	.801				

Note. Displayed are fixed effects of the full models including random slopes for all predictors.

Supplementary Table S7

Comparison of models using different measures of object specificity and frequency to predict basic-level scene categorization accuracy in Experiment 4, either with usage frequency as an additional predictor or without.

With usage frequency				Without usage frequency			
Model	AICc	Δ AICc	Weight	Model	AICc	Δ AICc	Weight
M6	4266.45	0	1	M6	4270.46	0	1
M5	4385.25	118.80	0	M5	4455.93	185.47	0
M8	4548.55	282.09	0	M8	4626.46	356.00	0
M9	4582.39	315.93	0	M7	4654.57	384.11	0
M7	4582.75	316.30	0	M9	4660.75	390.29	0

Note. Displayed are AICc values comparing non-nested models predicting basic-level scene categorization accuracy by different measures of object specificity and frequency (and usage frequency in the left models). Note the different ranking of models M7 and M9 in the two comparisons. M5: Averaged ratings, M6: Human ratings, M7: Own stimuli, M8: ADE subset, M9: ADE full database. See text for details.
